# A ferritin-like protein with antioxidant activity in *Ureaplasma urealyticum*

**DOI:** 10.1186/s12866-015-0485-6

**Published:** 2015-07-26

**Authors:** Guozhi Dai, Ranhui Li, Hongliang Chen, Chuanhao Jiang, Xiaoxing You, Yimou Wu

**Affiliations:** Institute of Pathogenic Biology, University of South China, Changsheng road 28, Hengyang, 421001 Hunan China; Hunan Province Cooperative Innovation Center for Molecular Target New Drug Study, Hengyang, 421001 Hunan China; Department of Clinical Laboratory, The First People’s Hospital of Chenzhou, Chenzhou, 42300 Hunan China

**Keywords:** *Ureaplasma urealyticum*, Ferritin, Reactive oxygen species, Antioxidant activity

## Abstract

**Background:**

*Ureaplasma urealyticum *is a major pathogen associated with many diseases. The ability of *U. urealyticum* to protect itself from oxidative stress is likely to be important for its pathogenesis and survival, but its oxidative stress tolerance mechanisms remain unclear. This study investigates the antioxidant activity of a ferritin-like protein from *U. urealyticum*.

**Results:**

The *uuferritin* gene, which was up regulated when *U. urealyticum* was subjected to oxidative stress, was cloned from *U. urealyticum* and the corresponding recombinant protein uuferritin was purified. Uuferritin protein reduced the levels of hydroxyl radicals generated by the Fenton reaction as a consequence of its ferroxidase activity, and thus the protein protected DNA from oxidative damage. Furthermore, oxidation-sensitive *Escherichia coli* mutants transformed with pTrc99a-uuferritin showed significantly improved tolerance to oxidative stress compared to *E. coli* mutants transformed with an empty pTrc99a vector.

**Conclusions:**

The present work shows that uuferritin protein confers resistance to oxidative stress *in vitro* and in *E. coli*. The protective role of uuferritin provides a foundation for understanding the mechanisms of oxidative stress tolerance in *U. urealyticum*.

## Background

*Ureaplasma urealyticum* is one of the smallest self-propagating prokaryotes; it lacks a cell wall, hydrolyzes urea to generate ATP, and belongs to the class Mollicutes [1]. In the adult female genital tract, *U. urealyticum* is a commensal and is sometimes considered to have low virulence [2]. However, *U. urealyticum* colonization has been associated with many diseases including brain abscess, urethritis, prostatitis, rheumatoid arthritis, and pelvic inflammation [2–6]. A *U. urealyticum* infection either in semen or in the female upper genital tract during pregnancy can lead to adverse pregnancy outcomes [6, 7]. It is still unclear how *U. urealyticum* affects the sperm and some researchers have found that *U. urealyticum* infection has no influence on sperm quality. However, several abnormal sperm characteristics have been reported in connection with *U. urealyticum* infections such as tail defects, decreased motility, altered morphology, and elevated levels of reactive oxygen species (ROS) [8–12]. The presence of *U. urealyticum* in placental parenchyma can lead to redox imbalance and to increased iron (Fe) concentrations, which have been related to an increased risk of preterm labor, fetal loss, and intraventricular hemorrhage [13, 14].

ROS are a by-product of normal metabolism and can be produced by the host phagocytic cells that constitute part of the human immune defense against invading pathogens [15]. In order to survive, bacteria have developed several mechanisms to combat the stress associated with ROS, including an up regulated enzyme system to repair damaged DNA or to rapidly detoxify the ROS. For example, some enzymes such as superoxidase dismutase (SOD), organic hydroperoxide resistance protein (Ohr), catalase (Kat), and alkyl hydroperoxide reductase (Ahpc) can detoxify ROS by reducing them to their corresponding alcohols [16–18]. These oxidative stress-related genes are usually up regulated by special transcription factors such as redox responsive Lys R-type regulator (OxyR), SoxRs, or ferric uptake regulator (Fur) [18–20].

The Fe ion is a cofactor for many enzymes and thus is involved in numerous physiological functions. On the other hand, in the ferrous form (Fe^2+^), Fe can react with hydrogen peroxide (H_2_O_2_) to generate ROS via the Fenton reaction, which may lead to metabolic dysfunction and become a major threat to cell survival under oxidative conditions [21]. Bacteria possess proteins of the ferritin superfamily, which are important for protection against oxidative stress [22–27]. The ferroxidase activity of ferritin is able to oxidize ferrous ions to the nonreactive ferric state. Ferritin proteins form a spherical protein complex in which a maximum of 4500 Fe^3+^ ions can be stored in a mineral form. It has been reported that ferritin proteins have the ability to prevent DNA damage through their ferroxidase activity by reducing the formation of hydroxyl radicals. Furthermore, some ferritin proteins can directly bind to DNA to protect it from oxidative damage [24, 26, 27].

Oxygen causes *U. urealyticum* persistence in the lungs of newborn mice, which potentiates the inflammatory response and turns a self-limited pneumonia into a lethal disease [28]. In addition, *U. urealyticum* elevates levels of ROS in sperm and endothelial cells, and stimulates macrophages to produce nitric oxidewhichacts in concert with ROS to inhibit the growth of *U. urealyticum* [13, 28, 29]. Patients with *U. urealyticum* have significantly higher ROS levels than those without *U. urealyticum*, implying that this bacterium confronts oxidative stress during colonization [13, 14]. Thus, the ability of *U. urealyticum* to protect itself from oxidative stress is likely to be important for its pathogenesis and survival. Genes encoding antioxidant enzymes like SOD, Kat, and AhpC are absent from the genome of *U. urealyticum*, but it does contain a gene for a ferritin-like (uuferritin) protein homolog, although its function is unclear [1].

In the current study, uuferritin transcript levels in *U. urealyticum* were dramatically increased after treatment with oxidants. Uuferritin protein suppressed the generation of hydroxyl radicals via the Fenton reaction and protected DNA by directly binding to it *in vitro*. In addition, uuferritin enhanced the tolerance of *E. oli* to oxidative stress. To our knowledge, this is the first experimental evidence that a *U. urealyticum* protein shows antioxidant activity.

## Methods

### *U. urealyticum* culture and oxidative stress treatment

*Ureaplasma urealyticum* (serovar 10 str. ATCC 33699) was cultured at 37 °C in 150 mL Ureaplasma broth medium containing 22.5 g mycoplasma broth base/L; 16.5 % horse serum; a 7.5 % solution of 25 % fresh yeast extract, 0.36 % urea, 380,000 U/L penicillin G; and phenol red according to Li et al. [29]. *U. urealyticum* was grown to early exponential phase as determined by color-changing units according to Li et al. [29]. To different flasks we added H_2_O_2_ (0.5 %), 2 mM cumene hydroperoxide (CHP), and 4 mM *tert*-butyl hydroperoxide (t-BHP), and all flasks were incubated at 37 °C for 20, 40, or 60 min.

### Real-time quantitative reverse transcription polymerase chain reaction (qRT-PCR)

Total RNA was extracted from *U. urealyticum* using Trizol reagent (Invitrogen, Carlsbad, CA, USA) according to the manufacturer’s instructions. After treatment with Dnase, 2 μg RNA was used for the first-strand cDNA synthesis (in a 20 μL reaction) using the SuperScript™ First-Strand Synthesis System for RT-PCR (Invitrogen, Carlsbad, CA, USA) according to the manufacturer’s instructions. The RT product (1 μL) was used as a PCR template to perform qRT-PCR in the ABI 7300 Real Time PCR System using SYBR® Premix Ex Taq™ (TaKaRa, Dalian, China) following the manufacturer’s instructions with 16S rRNA as an internal control. Quadruple reactions were conducted. Each experiment was repeated 3 times and consistent results were obtained. The relative mRNA expression level was calculated and statistically analyzed using the delta-delta-Ct method and *U*-test respectively, with non-treated samples as control.

### Cloning, expression, and purification

Genomic DNA was extracted from *U. urealyticum* using a MiniBEST Bacterial Genomic DNA Extraction Kit (TaKaRa) according to the manufacturer’s instructions. PCR primers were designed to amplify the *uuferritin* gene (NCBI accession no. WP_004025754) from *U. urealyticum*. The primers used in this study are listed in Table 1. The resulting PCR product was purified with a PCR purification kit (TaKaRa) and ligated into pMT18-T (TaKaRa). Because *U. urealyticum* has a nonstandard genetic code, TGA codons in the cloned sequence, which encode the amino acid tryptophan in *U. urealyticum* but would specify a stop codon in *E. coli*, were mutated to TGG using a TaKaRa MutanBEST Kit and the primer listed in Table 1 according to the manufacturer’s instructions. The resulting uuferritin coding sequences were digested with *Nde*I and *Bam*HI restriction endonucleases and ligated into pET-28a to generate the pET28a-uuferritin plasmid, which was transformed into *E. coli* Top10. The cloned sequence was confirmed by DNA sequencing (Invitrogen). Next, *E. coli* BL21 (DE3) (Novagen) was transformed with the pET28a-uuferritin plasmid and cultured in liquid medium containing 1 % (W/V) tryptone, 0.5 % (W/V) yeast extract, and 0.5 % (W/V) NaCl at 37 °C, and shaken at 200 rpm until the OD_600_ reached 0.5. The bacteria were then treated for 4 h with isopropyl-β-D-thiogalactopyranoside (IPTG) at a final concentration of 0.5 mM at 28 °C. The bacterial cells were collected and suspended in a sonication buffer containing 50 mM Tris–HCl, 50 mM NaCl, and 1 mM DL-Dithiothreitol (DTT), pH 8.0. The supernatant containing the uuferritin protein was applied to a chelating sepharose Fast Flow affinity column for purification (GE Healthcare, Shanghai, China). The column was washed with 50 mM Tris–HCl, 50 mM NaCl, and 40 mM imidazole, pH 8.0, and the uuferritin protein was eluted with 50 mM Tris–HCl, 50 mM NaCl, and 250 mM imidazole, pH 8.0.

**Table 1:** Oligonucleotide primers used in this study

Primer	Sequence (5′ → 3′)	Characteristic	Function
Fer-F	AAGGTATGCTTAGAAGAAGGTG		Real-time RT-PCR Evaluation of *uuferritin*
Fer-R	TTGTACGAACATCATCAAAATC		
16S-F	CAAGAATGAAACTCAAACGGAA		Real-time RT-PCR Evaluation of 16 s RNA (normalizer)
16S-R	CAACCATGCACCACCTGTC		
F-1	GTACATATGCAAGAGAAACCCC	*Nde*I	To amplify gene *uuferritin*
R-1	ACGGATCCTTATTTCTTGGAATATGGAGC	*Bam*HI	
MF-1	TTTGTAGATGATGGTATTAAAGATT		To mutate gene *uuferritin*
MR-1	CCATTTAACGAAACTAAAAGTTCA		

The underlined sequences are the restriction sites

### Intrinsic fluorescence assays

Samples (1 mL) containing 4 μM uuferritin in 50 mM 3-morpholinopropane-1-sulfonic acid (MOPS)-NaOH, pH 7.4, with and without 200 μM FeSO_4_ were incubated at 37 °C for 10 min. For measurements of intrinsic fluorescence, an excitation wavelength of 280 nm was used, and the fluorescence emission wavelengths were recorded from 300 to 400 nm with a F-4500 fluorescence instrument (Hitachi, Tokyo).

### Immobilized metal ion affinity chromatography experiments

The interaction between uuferritin and Fe^2+^ was assessed via immobilized metal ion affinity chromatography (IMAC) using a HiTrap Chelating HP 5-mL column (Amersham Pharmacia Biotech, Tokyo) according to Liu et al. [30]. The column was charged by applying 10 mL of a 100 mM solution of FeSO_4_. After washing out the excess metal ions with a 50 mM Tris buffer pH 7.4 (EQ buffer), 1 mL of uuferritin protein solution (1.5 mg/mL) was introduced into the column. The unbound uuferritin protein was washed out with EQ buffer, and the bound uuferritin protein was eluted by the addition of 5 mL of 200 mM EDTA. Next, 3 mL of each eluent was fractionated and a 15 μL sample was subjected to SDS-PAGE. A HiTrap Chelating HP column that was not charged with FeSO_4_ was used as a negative control.

### Fe mineralization

A 10 mM Fe^2+^ stock solution was freshly prepared by dissolving FeSO_4_ at pH 3.5. The final concentrations in the assays were: 0.5 μM uuferritin and 200 μM FeSO_4_ in 50 mM MOPS-NaOH, at pH 7.4. The kinetics of Fe mineralization were monitored at 37 °C using a Hitachi spectrophotometer F-4500 that recorded changes in absorbance at 305 nm after initiating the reaction with 200 μM Fe^2+^.

### DNA binding and protection

The DNA binding assays were performed according to Ishikawa et al. [31] with modifications. Briefly, 500 ng plasmid pET32a (Novagen) DNA was added to the total 20 μL reaction mixture comprising 20 μM FeSO_4_ (or 100 μM H_2_O_2_), 5 μM uuferritin protein or BSA protein, and 50 mM MOPS-NaOH, pH 7.4, and then incubated at 37 °C for 10 min. The reaction mixture was electrophoresed on a 1 % agarose gel, and the DNA in the gel was visualized and photographed under ultraviolet light after ethidium bromide staining.

DNA protection assays against hydroxyl radicals were performed under similar conditions to the DNA binding assays. A fresh solution of FeSO_4_ was added to obtain a final Fe concentration of 20 μM in a solution containing 5 μM uuferritin or BSA and pET32a (Novagen) DNA. After 10 min at 37 °C, the reaction mixtures received H_2_O_2_ at a final concentration of 100 μM in order to generate hydroxyl radicals via the Fenton reaction. The reactions were quenched after 10 min by adding an Fe chelator, EDTA, to a final concentration of 5 mM. The uuferritin protein was degraded by treating the solution with 20 μg/mL Pronasek (Sigma) for 30 min. The integrity of the DNA was analyzed by 1 % agarose gel electrophoresis.

### Antioxidant assay using oxidation of DCFH by Fenton reaction

The antioxidant activity of uuferritin was assayed by the fluorescence produced from the oxidation of 2, 7-dichlorodihydrofluorescein (DCFH) by the Fenton reaction according to Ko et al. [32] with modifications. Briefly, 50 μM DCFH was mixed with 100 μM H_2_O_2_, 20 μM FeSO_4_, and 50 mM MOPS-NaOH, pH 7.4 in the absence or presence of various amounts of uuferritin or BSA in 96-well plates. The total volume was 200 μL. The reaction was then started by adding 100 μM H_2_O_2_, maintained at 37 °C for 10 min, and then the fluorescence was measured using a spectrofluorimeter (SynergyHT, bio-TeK) at 485 nm excitation and 528 nm emission. The antioxidant activities were determined by the amount of uuferritin or BSA required for 50 % inhibition (IC_50_).

### Oxidative stress tolerance assay on pTrc99a-Uuferritin transformed ΔDps *E. coli* mutants

The *E. coli* Δ*dps* mutant strain JW0797 (the *dps* single-gene-knockout strain), derived from the parent strain BW25113 [*rrnB*3 Δ*lacZ4787 hsdR514*Δ(*araBAD*) *567*Δ(*rhaBAD*) *568 rph-1*], was used for the oxidative stress-resistance assay. These *E. coli* strains originated from the Nara Institute of Science and Technology (Ikoma, Nara, Japan) [33]. The *uuferritin* gene was double-digested by *Nco*1 and *Bam*H1 from pET28a-uuferritin and then inserted into plasmid pTrc99a to generate pTrc99a-uuferritin. Both pTrc99a and pTrc99a-Uuferritin were transformed into *E. coli* JW0797 for the oxidative stress-resistance assay. Antibiotics were used at final concentrations of 50 μg/mL ampicillin and 50 μg/mL kanamycin. The *E. coli* cells were grown overnight in Luria-Bertani (LB) broth with aeration at 37 °C and shaken at 200 rpm. The cultures were induced with IPTG at a final concentration of 0.5 mM for 4 h and then exposed to oxidative stress (30 mM H_2_O_2_ or 25 mM FeSO_4_) for 3 h. The concentrations of these cultures were identified at OD_600_ = 0.8 and then diluted serially (1:10, 1:100, or 1:1000). Each sample (10 μL) was spotted onto LB plates and cultured overnight at 37 °C. In addition, 100 μL of stressed and unstressed samples were spread separately on the LB plates. The number of colonies on each plate was recorded after the plates were incubated at 37 °C for 16 h. Survival ratios of the bacteria under oxidative conditions were calculated according to the following formula: survival ratio = (colony number after oxidative stress/colony number of unstressed bacteria) × 100 %. The experiments were repeated 5 times. The survival ratios and standard deviations are shown in Fig. 4.

### Statistical analysis

To compare the relative transcriptional profiles of the candidate genes statistical comparisons were made using one-way analysis of variance and Student’s *t*-test. Differences at *P* values < 0.05 were considered to be statically significant at the 95 % confidence level. Data were expressed as mean ± standard deviation (SD) from at least 3 independent experiments.

## Results

### The *Uuferritin* gene is upregulated by oxidative stress

**Fig. 1:**
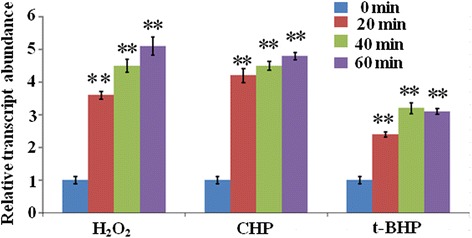
Quantitative real-time PCR analysis of *uuferritin* expression under H_2_O_2_, CHP, and t-BHP stress conditions. The samples were collected at 0, 20, 40, and 60 min after treatment. **p < 0.01 by *U*-test. Bars represent SD (*n* = 3)

Transcripts of the ferritin superfamily have been reported to be upregulated under oxidative stress conditions [22, 34]. To confirm whether the expression of *uuferritin* is affected by oxidative stress, qRT-PCR assays were performed. *Uuferritin* transcription was significantly increased at 20, 40, and 60 min when treated with H_2_O_2_ (3.5-, 4.4-, and 5.1-fold, respectively), CHP (4.4-, 4.5-, and 4.7-fold, respectively), or t-BHP (2.4-, 3.2-, and 3.1-fold, respectively), indicating that *uuferritin* is associated with oxidative stress tolerance in *U. urealyticum* (Fig. 1). In response to oxidative stress, the expression of ferritin proteins is regulated by Fur, PerR (peroxide stress response regulator), or OxyR [17, 19]. However, the *U. urealyticum* genome sequence exhibits no known sequences for genes that are related to these regulators [1]. The mechanism of *U. urealyticum* response to oxidative stress and the regulation of uuferritin therefore remains unclear and requires further study.

### Ferroxidase activity of Uuferritin

**Fig. 2:**
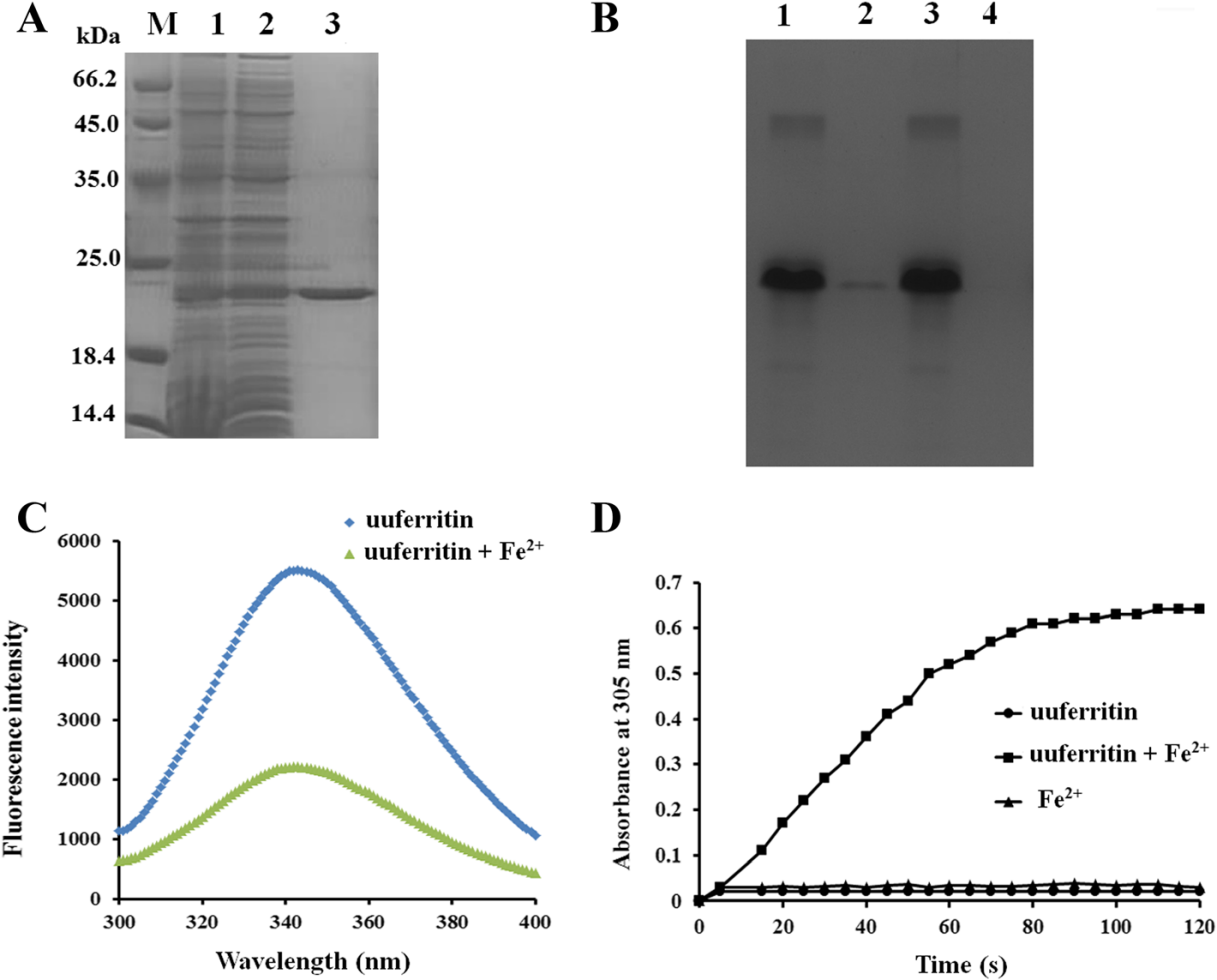
Ferroxidase activity of uuferritin. **a** The expression and purification of recombinant uuferritin protein. M: Molecular weight marker; Lane 1 and 2: The uuferritin protein induced by IPTG; Lane 3: The uuferritin protein after purification through a Ni^2+^ column. **b** The Fe^2+^ binding characteristics of uuferritin were investigated using IMAC. Lane 1: The columns were charged with Fe^2+^ and the bound protein was eluted with EDTA. Lane 2: The columns were charged with Fe^2+^ and the bound protein was eluted with EQ buffer. Lane 3: The column was not charged with metal and was eluted with EQ buffer. Lane 4: The column was not charged with metal and was eluted with EDTA. The samples were subjected to SDS-PAGE and stained with Coomassie Brilliant Blue. **c** The intrinsic fluorescence of 4 μM uuferritin protein was detected when 200 μM FeSO_4_ was added. **d** Spectrophotometric kinetic curve of Fe^2+^ oxidation by uuferritin. Reactions were performed in 50 mM MOPS-NaOH, pH 7.4, at 37 °C. Reactions were started by the addition of 100 μM FeSO_4_; the formation of Fe core was monitored by measuring absorbance at 305 nm in a 0.5 cm cuvette. Symbols for conditions are as follows: squares, 0.5 μM uuferritin and Fe^2+^; circles, 0.5 μM uuferritin only; triangles, Fe^2+^ only

To determine whether the *uuferritin* gene encodes a functional ferritin protein, the open reading frame (ORF) corresponding to the *U. urealyticum* ferritin protein (uuferritin) was analyzed. The complete ORF was identified by sequencing, and cloned into the expression vector pET28a to generate plasmid pET28a-uuferritin, which encodes the N-terminal His-tagged protein. Upon IPTG induction, *E. coli* BL21 (DE3) cells transformed with plasmid pET28a-uuferritin expressed a soluble protein with the expected molecular mass of 22 kDa which was identical to the theoretical molecular mass of His-tagged uuferritin. The overexpressed His-tagged uuferritin protein was purified by Ni^2+^-affinity chromatography (Fig. 2a). Following purification, the Fe^2+^ binding characteristics of uuferritin were investigated using IMAC. The uuferritin protein was not retained in the column not charged with Fe^2+^. However, it did remain in the column with immobilized Fe^2+^. The proteins retained in these columns were eluted by EDTA, indicating that uuferritin was retained in the columns due to Fe^2+^ binding (Fig. 2b).

The intrinsic fluorescence of a protein is affected by aromatic residues and its conformational state, and the fluorescence can be quenched by directly metal binding [30]. To determine the binding property of uuferritin protein to Fe^2+^, intrinsic fluorescence quenching of uuferritin by Fe^2+^ binding was measured. The results show that addition of Fe^2+^ dramatically quenched the intrinsic fluorescence of uuferritin, which demonstrates that uuferritin binds directly to Fe^2+^ (Fig. 2c).

Spectral analysis at 305 nm was used to monitor the ferroxidase activity of ferritin, which converts Fe^2+^ to Fe^3+^. Our results show that when uuferritin was incubated with FeSO_4_, there was a rapid increase in absorbance at 305 nm, whereas in control experiments without uuferritin, negligible changes in absorbance of a FeSO_4_ solution were detected (Fig. 2d). Our results indicate that the uuferritin protein, like other members of the ferritin superfamily from mycobacteria, *E. coli* or *Bacteroides fragilis*, has ferroxidase activity that converts Fe^2+^ into Fe^3+^ and then sequesters Fe by storing it as a mineral inside a protein cage [23, 24, 26].

### Antioxidant activity of uuferritin

**Fig. 3:**
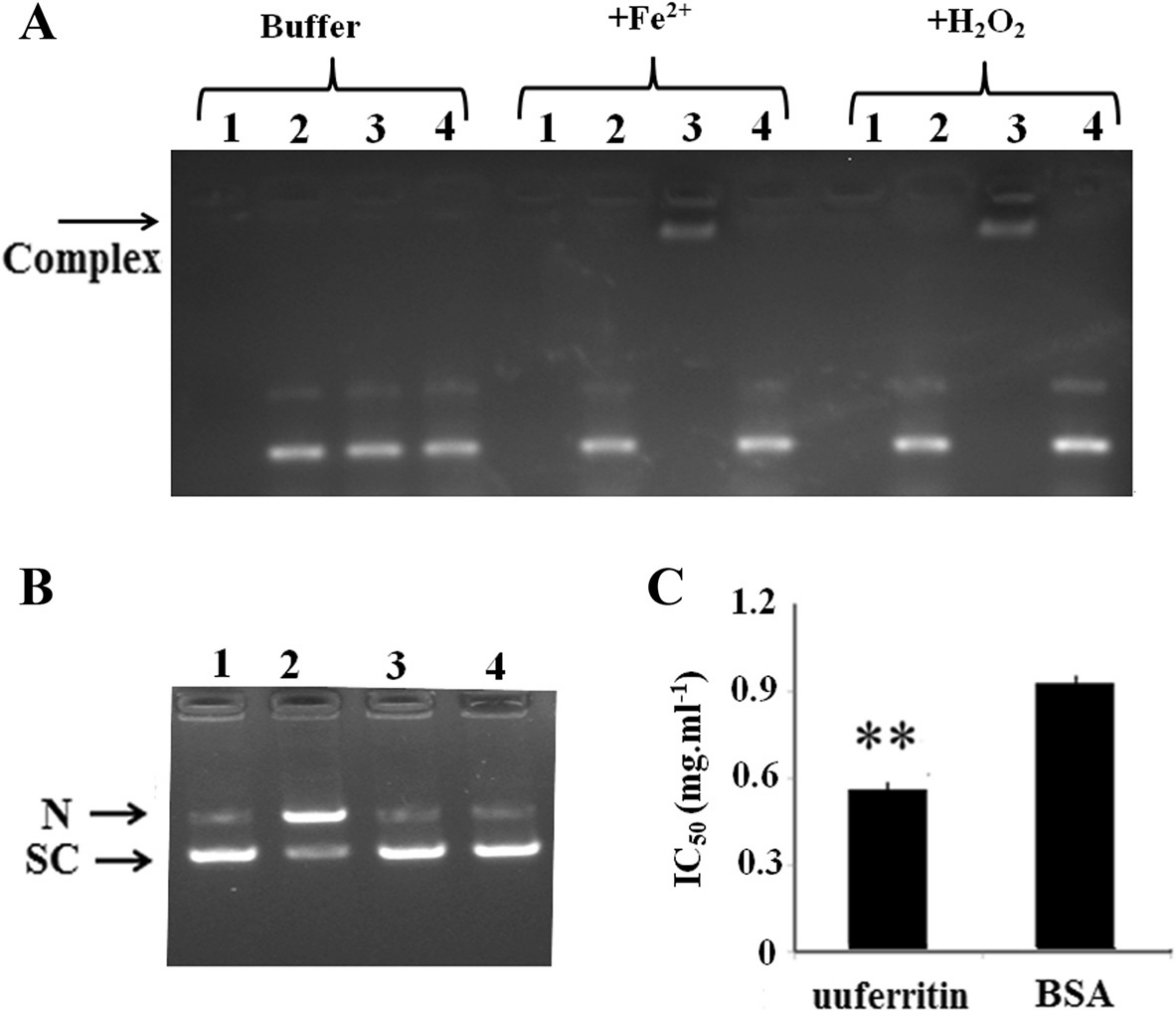
The antioxidative activity of uuferritin *in vitro*. **a** The DNA binding activity of uuferritin is promoted by either Fe^2+^ or H_2_O_2_. The DNA binding activity of uuferritin was analyzed by the capacity to retard the migration of supercoiled pET32a plasmid in 1 % agarose gel. The DNA on the gel was stained with ethidium bromide. Binding reactions were performed in 50 mM MOPS-NaOH, pH7.4, containing 20 μM Fe^2+^or 100 μM H_2_O_2_ as indicated. Lanes: 1, Uuferritin only; 2, DNA only; 3, uuferritin plus DNA; 4, BSA plus DNA. The uuferritin-DNA complexes are indicated by the arrow. **b** The uuferritin protein protects DNA from hydroxyl radicals. Supercoiled pET32a plasmid was incubated with BSA (lanes 1 and 2) or with uuferritin (lanes 3 and 4) in 50 mM MOPS-NaOH, pH7.4, for 10 min at 25 °C. Hydroxyl radical generation via the Fenton reaction was achieved by adding 20 μM FeSO_4_ and 100 μM H_2_O_2_ (lanes 2 and 4). Reactions were quenched and the uuferritin protein was degraded by Pronasek. Then DNA was analyzed on an agarose gel stained with ethidium bromide. (SC: supercoil DNA, N: nicked DNA). **c** The uuferritin suppressed the generation of the hydroxyl radical via the Fenton reaction. **p < 0.01 by *U*-test. Bars represent SD (*n* = 3)

The ability of uuferritin to interact with supercoiled plasmid DNA was analyzed by electrophoretic mobility shift assay. When uuferritin was incubated with DNA, there were no changes in DNA mobility through agarose gel electrophoresis (Fig. 3a). However, when Fe^2+^ or H_2_O_2_ were introduced, the DNA did not enter the gel, which suggests that DNA may interact with uuferritin to form a uuferritin-DNA complex (Fig. 3a). The ability of uuferritin to interact with DNA suggests that it could protect DNA from oxidative damage. Hence, we examined the ability of uuferritin to protect plasmid DNA from hydroxyl radicals. The combination of Fe^2+^ and H_2_O_2_ leads to the formation of hydroxyl radicals, which in turn causes double-stranded DNA breaks that convert supercoiled circular plasmid DNA to linear DNA and thus can be detected by electrophoresis. When plasmid DNA (pET32) was treated with Fe^2+^ and H_2_O_2_, it was converted to nicked DNA, suggesting the occurrence of double-stranded DNA breaks (Fig. 3b). However, when uuferritin was present, the plasmid DNA remained unchanged after being treated by the oxidative stress, suggesting that the formation of a protein-DNA complex could potentially protect the DNA from oxidative damage (Fig. 3b).

Hydroxyl radicals are highly active and can be generated by the Fe-promoted Fenton reaction. To determine whether the uuferritin protein can inhibit hydroxyl radical generation, the effect of uuferritin on hydroxyl radicals generated by Fe^2+^ was analyzed by measuring its ability to inhibit the fluorescence produced from DCFH oxidized by the Fenton reagent. The antioxidant activity of uuferritin protein was found to be better than that of BSA, a standard hydroxyl radical scavenger (Fig. 3c). Thus, these results indicate that uuferritin protein could reduce the level of hydroxyl radicals generated by the Fenton reaction as a consequence of its ferroxidase activity.

### A pTrc99a-uuferritin transformed *E. coli* Dps mutant shows improved tolerance of oxidative stress and Fe^2+^

**Fig. 4:**
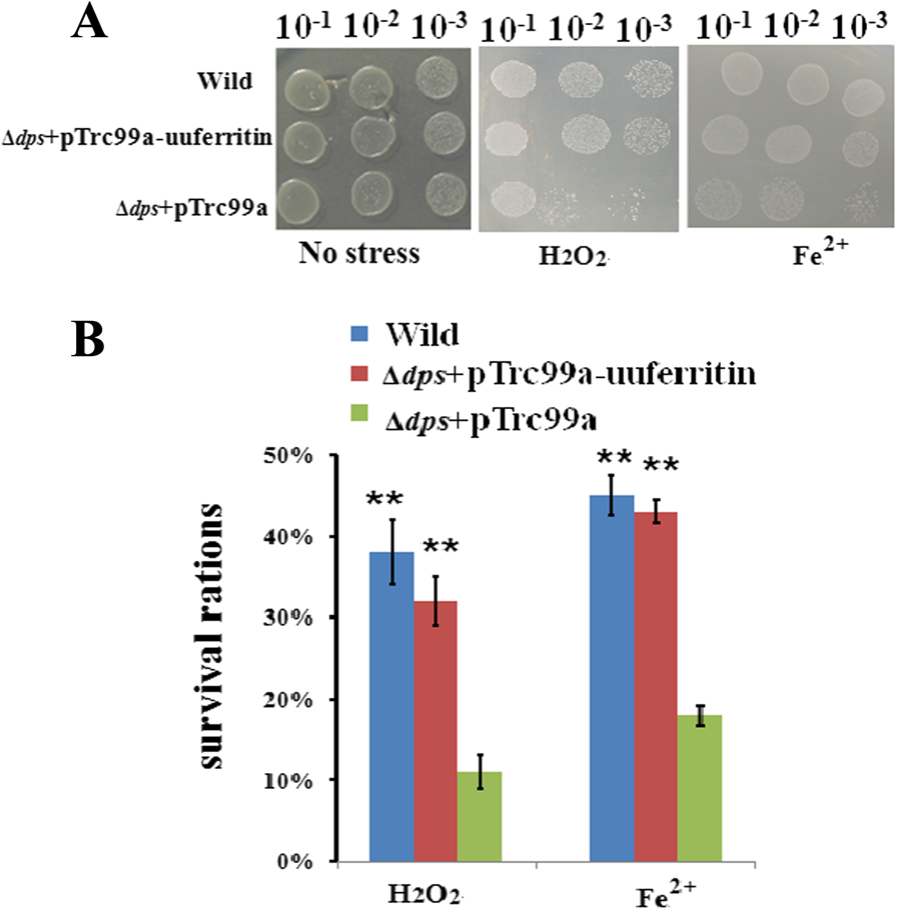
The *uuferritin* gene complements the *E. coli* Δ*dps* mutant in the assay of colony patches (**a**) and survival ratio (**b**). The presence of the *uuferritin* gene increased the survival of the *E. coli* Δ*dps* mutant strain under the 30 mM H_2_O_2_ or 25 mM Fe^2+^ stress, but the pTrc99a alone had no effect on survival. **p < 0.01 by *U*-test. Bars represent SD (*n* = 3)

*E. coli* has been successfully used as a model to explore the functions of bacterial ferritin proteins, including their antioxidant activity [34, 35]. Here, the *E. coli* oxidation-sensitive mutant Δ*dps* JW0797-1 was transformed using plasmid pTrc99a-uuferritin to assess the effect of uuferritin on oxidative stress. A spot assay showed that the number of colonies growing on agar plates was much greater for pTrc99a-uuferritin transformed Δ*dps E. coli* than for the corresponding pTrc99a-transformed control when the cells were subjected to H_2_O_2_ or FeSO_4_ (Fig. 4a). Furthermore, when exposed to 30 mM H_2_O_2_ or 25 mM FeSO_4_, the survival ratios of pTrc99a-Uuferritin transformed Δ*dps E. coli* were about 38 % and 40 %, respectively, which were much higher than 11 % and 18 %, respectively, for the control (Fig. 4b).

We tried to transform the *E. coli* BL21 strain using pET28a-uuferritin, although the difference in oxidative stress resistance between pET28a vector-transformed BL21 control and pET28a-uuferritin-transformed BL21 was not significant (data not shown). Hence, we tried to transform the oxidation-sensitive *E. coli* deletion mutant Δ*dps.* The Dps proteins belong to the ferritin protein superfamily and play significant roles in tolerance to Fe^2+^ and H_2_O_2_. It has been reported that the growth of Δ*dps* strains of *E. coli* is arrested by hydrogen peroxide and metal stress [36]. The pTrc99a-uuferritin-transformed Δ*dps* mutant showed greater tolerance of oxidative stress than the pTrc99a-vector-transformed Δ*dps* control, suggesting that uuferritin has antioxidant activity.

## Discussion

Bacteria are known for their unique ability to adapt to varying life styles and environments, even under adverse conditions. Bacteria can produce H_2_O_2_ to inhibit the growth of other bacteria, which gives them a competitive advantage [15]. Part of the human host defense against pathogen infection is the production of ROS that can kill invading bacteria [15]. Some antioxidants, such as SOD or Ahpc, which are highly conserved in other bacteria, are absent in mycoplasmas, which are the smallest and simplest self-replicating organisms, because of their small genome size [1, 37]. However, enzymes such as a peroxidase [38], or the Omc/Ohr and MrsA proteins, have been proposed to protect mycoplasmas against oxidative stress [16, 39]. In the current paper, we found that uuferritin exhibited antioxidant activity *in vitro* and in *E. coli*, indicating that this protein might protect *U. urealyticum* from the ROS that are released from human phagocytic cells and other bacteria.

The Fe in living organisms is a “double-edged sword”: it is a critical nutrient for the growth and survival of most bacterial species, but it is also potentially harmful [40]. The free Fe ion can act catalytically via the Fenton reaction to produce hydroxyl radicals that damage lipids, proteins, and DNA [40]; as a result, free intracellular Fe must be maintained at low levels [21]. The proteins of the ferritin superfamily, which include ferritin and Dps along with bacterioferritin, are defined by their ferroxidase activity and their ability to bind Fe; they are distributed across all three domains of life [41, 42]. The ferritins can convert Fe^2+^ to Fe^3+^ and then store the Fe in a nonreactive mineral form, Fe_2_O_3_, inside a protein cage. We found that the ferroxidase activity of uuferritin protein allowed it to inhibit the production of hydroxyl radicals *in vitro*. For this reason, we speculate that uuferritin can help to maintain Fe homeostasis, reduce Fe toxicity, and prevent oxidative damage by storing excess free Fe in *U. urealyticum.*

The exposure of DNA to ROS can generate a battery of single nucleobases and bulky DNA lesions. Bacteria have evolved various mechanisms to protect their DNA from oxidative stress [43], including the upregulation of many DNA repair proteins in response to ROS-induced DNA damage [43, 44]. Some Dps proteins, such as those from *E. coli* and *Candidatus Legionella jeonii*, are able to physically protect DNA by the formation of nonspecific protein-DNA complexes [27, 45]. Other Dps proteins, such as *Deinococcus radiodurans* Dps-2 [46], *H. pylori* NapA [47], *Agrobacterium tumefaciens* Dps [48], and *Campylobacter jejuni* Dps [35], have been reported to have no DNA-binding ability under normal conditions. However, *C. jejuni* Dps was able to bind DNA in response to Fe^2+^ or H_2_O_2_ [35], while Dps-DNA binding was promoted by Fe^2+^ in *H. pylori* [47]. The amino acid sequences of uuferritin share very little similarity with *C. jejuni* Dps, *H. pylori* NapA proteins or *E. coli* bacterioferritin while it has a ferroxidase diiron center motif which is conservative in ferritin superfamily. The uuferritin showed certain homology with bacterial ferritin protein from *Thermotoga maritima* [49]. So we speculate the uufferritin belongs to the bacterial ferritin. Nevertheless, like *C. jejuni* Dps and *H. pylori* NapA, uuferritin did not bind DNA under normal conditions, but could do so on exposure to Fe^2+^ or H_2_O_2_. How Fe^2+^ or H_2_O_2_ stimulate uuferritin binding to DNA remains unclear and requires further research.

Mutagenesis has been successfully applied to some mycoplasmas to disrupt nonessential genes, but similar approaches in *U. urealyticum* have not yet been successful [50]. Furthermore, the tools for exploring the functions of *U. urealyticum* proteins are limited. In this paper, a heterologous *E. coli* expression system was used to show that the uuferritin protein can function as an antioxidant and provide cellular protection from external oxidative challenge. The strategy was based on the observation that *E. coli* cells expressing *ferritin* like genes are capable of tolerating higher levels of oxidative stress than control cultures [31, 34]. In the present study, the ability of *E. coli* harboring the *uuferritin* gene to grow in the presence of H_2_O_2_ or Fe^2+^ was used as a functional assay to examine the putative function of uuferritin. Growth performance in media containing H_2_O_2_ or Fe^2+^ revealed that uuferritin has a protective function in *E. coli.* Therefore, we consider that the improved growth performance under oxidative stress was due to the function of the uuferritin protein.

## Conclusions

In the present paper, we demonstrate that the expression of *uuferritin* in *U. urealyticum* is elevated in response to oxidative stress. The uuferritin protein inhibits the Fenton reaction via its ferroxidase activity and protects DNA. In addition, uuferritin complements the deficiency in oxidative tolerance caused by *dps* deletion in *E. coli*. This study may prove valuable in understanding the antioxidant mechanisms of *U. urealyticum*.
